# Grazing exclusion is more effective for vegetation restoration and nutrient transfer in the heavily degraded desert steppe

**DOI:** 10.1186/s12870-024-05127-z

**Published:** 2024-05-17

**Authors:** Dongjie Hou, Jiayue Liu, Nan Li, Beilei Han, Changcheng Liu, Zhongwu Wang

**Affiliations:** 1 College of Grassland, Resource and Environment, Inner Mongolia Agricultural University, 306 Zhaowuda Road, Saihan District, Hohhot, 010019 China; 2 Institute of Grassland Research, Chinese Academy of Agricultural Science, Hohhot, 010020 China; 3 State Key Laboratory of Vegetation and Environment Change, Institute of Botany, Chinese Academy of Sciences, Beijing, 100093 China

**Keywords:** Desert steppe, Fencing, Grassland degradation stage, Plant nutrient, Soil nutrient

## Abstract

**Background:**

Grazing exclusion is an efficient practice to restore degraded grassland ecosystems by eliminating external disturbances and improving ecosystems’ self-healing capacities, which affects the ecological processes of soil-plant systems. Grassland degradation levels play a critical role in regulating these ecological processes. However, the effects of vegetation and soil states at different degradation stages on grassland ecosystem restoration are not fully understood. To better understand this, desert steppe at three levels of degradation (light, moderate, and heavy degradation) was fenced for 6 years in Inner Mongolia, China. Community characteristics were investigated, and nutrient concentrations of the soil (0–10 cm depth) and dominant plants were measured.

**Results:**

We found that grazing exclusion increased shoots’ carbon (C) concentrations, C/N, and C/P, but significantly decreased shoots’ nitrogen (N) and phosphorus (P) concentrations for *Stipa breviflora* and *Cleistogenes songorica*. Interestingly, there were no significant differences in nutrient concentrations of these two species among the three degraded desert steppes after grazing exclusion. After grazing exclusion, annual accumulation rates of aboveground C, N, and P pools in the heavily degraded area were the highest, but the aboveground nutrient pools were the lowest among the three degraded grasslands. Similarly, the annual recovery rates of community height, cover, and aboveground biomass in the heavily degraded desert steppe were the highest among the three degraded steppes after grazing exclusion. These results indicate that grazing exclusion is more effective for vegetation restoration in the heavily degraded desert steppe. The soil total carbon, total nitrogen, total phosphorus, available nitrogen, and available phosphorus concentrations in the moderately and heavily degraded desert steppes were significantly decreased after six years of grazing exclusion, whereas these were no changes in the lightly degraded desert steppe. Structural equation model analysis showed that the grassland degradation level mainly altered the community aboveground biomass and aboveground nutrient pool, driving the decrease in soil nutrient concentrations and accelerating nutrient transfer from soil to plant community, especially in the heavily degraded grassland.

**Conclusions:**

Our study emphasizes the importance of grassland degradation level in ecosystem restoration and provides theoretical guidance for scientific formulation of containment policies.

## Introduction

Carbon (C), nitrogen (N), and phosphorus (P) are crucial for plants’ growth and physiological metabolism in terrestrial ecosystems [1]. For example, C is an important component of plants, while N and P are involved in protein and nucleic acid synthesis. Plants can rapidly alter these nutrient concentrations and their stoichiometry to adapt to environmental changes [2]. Thus, C, N, P, and their stoichiometry are considered to be sensitive indicators of plants’ adaptations to changes in ecosystem structure and function [3]. Soil nutrient supply plays an important role in determining plant growth and ecosystem structure and function [4]. Plants absorb nutrients from the soil through their roots to maintain their physiological activities and alter their interspecies relations, which in turn dominate the changes in community structure and function [5]. Therefore, exploring the role of nutrients in the relationship between soil and plants is helpful to reveal the underlying mechanisms of ecological processes in terrestrial ecosystems from the perspective of nutrient transfer and utilization.

Grassland ecosystems are important terrestrial ecosystems in which N and P are limited, and they have critical ecological and economic functions [4, 6]. However, intense human activities, such as overgrazing and farming, have led to severe damage to the vegetation and soil of grasslands, resulting in ecosystem degradation and causing eco-environmental problems, such as decreased community productivity, biodiversity loss, dust storms, and soil erosion [7]. Grazing exclusion is an effective practice that has been widely applied in various degraded grassland ecosystems [8–10]. By eliminating the direct effects of external disturbances on a plant community and the soil, grazing exclusion uses the ecosystem’s self-healing capacities to drive the restoration and succession of degraded grasslands [11, 12]. During this period, changes in some ecological processes, such as the disappearance of livestock foraging, trampling, and fecal and urine return will alter soil and plant nutrient status at multiple levels, driving the vegetation restoration of grassland ecosystems [13, 14].

The nutrient relationship between soil and plants in fenced grasslands is a key topic in grassland management, as it is critical to understand the structural and functional restoration of fenced grassland ecosystems. Previous studies have reported conflicting results regarding the effects of grazing exclusion on soil and plant nutrients. For example, some studies have shown that grazing exclusion significantly increased soil and plant nutrient concentrations [10, 15], while others have found the opposite [14, 16, 17]. This uncertainty in how grazing exclusion affects soil and plant nutrients has led to some controversial results. For example, some studies found that grazing exclusion increased a plant community’s height, cover, and aboveground biomass [18, 19], but other studies have found that it decreased these traits [20, 21]. Differences in the duration of grazing exclusion, grassland type, and climate type account for differing results [21–24]. However, the degree of grassland degradation (i.e., light, moderate, or heavy degradation) before grazing exclusion began is another important factor that has been overlooked in previous research [3]. For example, lightly degraded grasslands usually have higher community aboveground biomass and soil nutrient concentrations. The structure and functions of grassland ecosystems could be restored rapidly after short-term grazing exclusion [3], increasing soil and plant nutrient concentrations and accelerating the nutrient cycle of grassland ecosystems. On the other hand, heavily degraded grasslands have lower community aboveground biomass and soil nutrient concentrations. The restoration of the plant community and soil nutrients in heavily degraded grasslands takes a long time. Thus, the ecological processes in differently degraded grasslands might have divergent responses after grazing exclusion [25]. Moreover, there is a lack of clear understanding of the nutrient relationships between plant-soil systems and vegetation restoration in differently degraded grasslands after grazing exclusion.

Desert steppe is widely distributed ecosystem in central and western Inner Mongolia, and it has important ecological functions [26]. However, the desert steppe in Inner Mongolia is facing ecosystem degradation caused by climate change and rapid increases in the numbers of livestock. A significant proportion of the desert steppe has had moderate or heavy degradation, and only a small proportion has had light or no degradation [27]. Due to low precipitation (150–250 mm), poor soil fertility, and strong solar radiation in the desert steppe, grazing exclusion is the main technique to restore these degraded grassland ecosystems [28, 29]. In this study, sections of desert steppe at different degradation levels (light, moderate, and heavy degradation) was selected for a research experiment and fenced for 6 years. This study aimed to explore (1) whether the nutrient concentrations of soil and plants and the plant community characteristics in desert steppe under different degraded stages had consistent responses to grazing exclusion, and (2) the relationship between vegetation restoration and nutrient transfer of a soil-plant system in differently degraded desert steppes after grazing exclusion. We hypothesized that (1) heavily degraded desert steppe would have the fastest vegetation recovery rate and the highest plant nutrients, but the worst vegetation state and the lowest soil nutrients after grazing exclusion. (2) In order to ensure nutrient balance between belowground and aboveground, vegetation restoration would accelerate nutrient transfer from the soil to the plant community, especially in the heavily degraded desert steppe. This study strengthens our understanding of the relationship between vegetation restoration and nutrient transfer in fenced grassland ecosystems and provides a theoretical basis for the restoration and management of degraded desert steppe and the assessment of fencing’s effects on nutrient utilization.

## Materials and methods

### Study site

This study was conducted in the field experiment site of Inner Mongolia Agricultural University (41°27′17″N, 111°53′46″E, altitude 1456 m) located in the Siziwang Banner of Ulanqab City, Inner Mongolia, China. The region has a temperate continental monsoon climate, characterized by cold and dry winter, and hot and moist summers. The mean annual temperature is 3.4℃, and the mean annual precipitation is about 220 mm, with 80% of this occurring in the growing season (June to September). The soil type is light brown calcium soil (the Chinese Soil Classification), with low soil-available nutrients. In this region, the vegetation is typical of a temperate desert steppe, with simple species composition and low productivity. The dominant species is *Stipa breviflora*, and other species include *Cleistogenes songorica*, *Convolvulus ammannii*, *Artemisia frigida*, *Salsola collina*, *Caragana stenophylla*, and *Kochia prostrata*.

### Experimental design

**Fig. 1:**
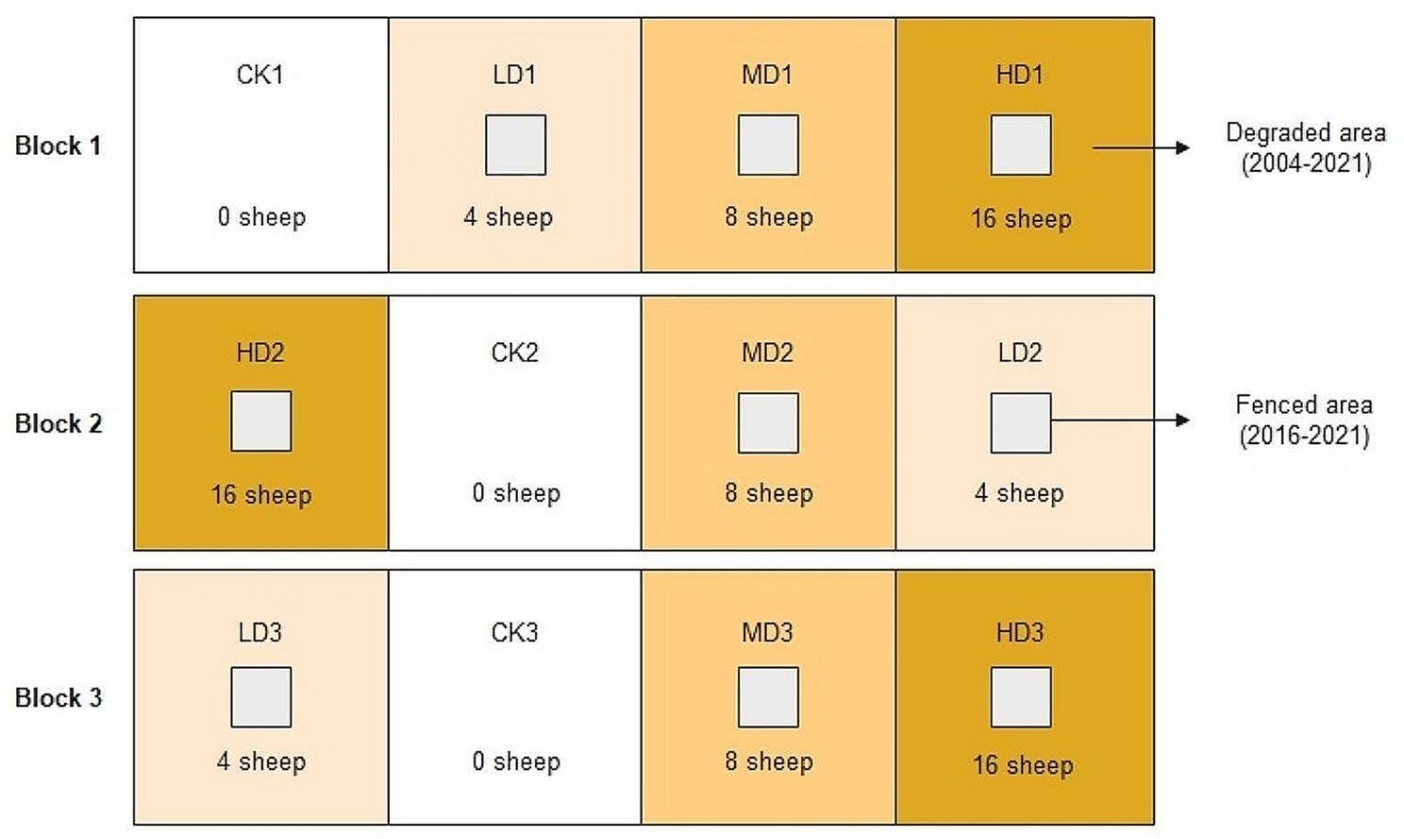
Diagram of the experimental design. LD: light degradation; MD: moderate degradation; HD: heavy degradation; CK: control; the gray square indicates the fenced area

The experiment site was created in 2004 using a randomized block design. Twelve grazing plots (three blocks of four plots each), each with an area of 4.4 hm^2^, were set up in a zone with uniform terrain and vegetation, and the three blocks were independent of each other (Fig. 1). The experimental treatments included a control treatment (0 sheep/plot), light grazing (4 sheep/plot), moderate grazing (8 sheep/plot), and heavy grazing (16 sheep/plot). The grazing intensity was set according to the actual grazing conditions of local herders. The four grazing intensities were calculated to be 0 sheep/hm^2^/year, 0.91 sheep/hm^2^/year, 1.82 sheep/hm^2^/year, and 2.71 sheep/hm^2^/year. The livestock used for grazing were two-year-old Mongolian sheep, and the grazing period was from early June to late October each year. By 2016, the grazing site had been used for 12 years. Based on the dividing method described by Liu et al., degradation levels were evaluated based on community cover and species composition [3]. The community cover was decreased by 10-30% in lightly degraded areas, 30-60% in moderately degraded areas, and 60-90% in heavily degraded areas. In our experimental site, the community cover gradually decreased and species composition shifted along with grazing intensity (Table 1).

Before the 2016 growing season, a 50 m × 50 m plot was selected in the center of each degraded plot and was enclosed with a net fence as grazing exclusion treatment (Fig. 1). All grazing exclusion plots were free from any disturbance after 2016. By 2021, the grazing exclusion plots had been fenced for 6 years, and the vegetation and soil had been restored. The outside of the fencing area was still treated according to the previous treatments. The vegetation status outside the fence (i.e., in the degraded area) in 2021 was similar to that in 2016 (Table 1). *Stipa breviflora* dominated the lightly degraded plots, whereas *Cleistogenes songorica* and some annual plants dominated the moderately and heavily degraded plots.

**Table 1:** Degradation information of experimental plots

Experimental treatments	Main species		Community cover (%)	
	2016	2021	2016	2021
Control treatment	*Stipa breviflora, Cleistogenes songorica, Artemisia frigida, Potentilla bifurca, Allium tenuissimum*,	*Stipa breviflora, Cleistogenes songorica, Artemisia frigida, Allium tenuissimum, Cymbaria mongolica, Potentilla bifurca*	45.4	42.7
Light degradation	*Stipa breviflora, Cleistogenes songorica, Convolvulus ammannii*,	*Stipa breviflora, Cleistogenes songorica, Convolvulus ammannii, Bassia prostrata*	32.5	30.8
Moderate degradation	*Stipa breviflora, Cleistogenes songorica, Chenopodium aristatum*	*Stipa breviflora, Cleistogenes songorica, Chenopodium aristatum, C. album, Caragana stenophylla*,	26.4	24.8
Heavy degradation	*Cleistogenes songorica, Stipa breviflora, Chenopodium aristatum*,	*Cleistogenes songorica, Stipa breviflora, Chenopodium aristatum, Euphorbia humifusa, Eragrostis minor*	15.1	13.2

*Note* Species are ranked according to their importance value in the plant community

### Sample collection and measurement

To collect plant community and soil samples, quadrat survey was used at the peak of community aboveground biomass (mid-August) in 2021. The centers of the four fenced enclosures were taken as reference points, and one 1 m × 1 m quadrat (4 quadrat/plot ×3 plot/treatment = 12 quadrat/treatment) was set up at a 5 m distance inside and outside the fence to avoid edge effects. In each quadrat, species composition was recorded, and community cover and species height were measured using visual estimation and a ruler (measuring three randomly selected individuals from each species), respectively. Finally, the aboveground part of each species was harvested into envelopes. After the plant community survey was completed, a soil auger with diameter of 3.8 cm was employed to collect 0–10 cm of soil depth at the center of each quadrat in each experimental plot.

Ten to fifteen mature and healthy plants of *S. breviflora* (dominant species) and *C. songorica* (subdominant species) were randomly selected in each plot, and the aboveground parts were collected with scissors, mixed evenly, put into envelopes, and brought back to the laboratory.

Plant samples were dried at 65℃ to a constant weight and then weighed. Roots and stones in soil samples were carefully removed, mixed evenly into envelopes, and dried in the laboratory. Part of the soil sample was used to measure the available N and P concentrations through a 2 mm sifter. The other was used to measure total carbon, total nitrogen, and total phosphorus concentrations through a 0.15 mm sifter.

Plant samples were cut into 1–2 cm pieces and ground with a mill (MM400, Retsch). The C and N concentrations of the plant and soil samples were determined using a Vario ELIII CHNOS Elemental Analyzer (Elementar Analysensysteme GmbH). The P concentrations of these samples were determined using an ICP-OES (iCAP 6300 ICP-OES Spectrometer, Thermo Scientific) after digestion. Available N and P concentrations were measured using the alkalolysis diffusion method and a UV spectrophotometer (UV-2550, UV-Visible Spectrophotometer, Shimadzu).

### Data analysis

In this region, more than 90% of the community aboveground biomass is composed of *S. breviflora* and *C. songorica*. The community aboveground nutrient pool is calculated as the sum of the product of aboveground biomass and nutrient concentration of the two species, as represented in the following equation [14]:

Community aboveground nutrient pool =$\sum _{1}^{i}{C}_{i}\times {B}_{i}$

where *Ci* and *Bi* indicate nutrient concentration and aboveground biomass of a species, respectively.

The annual vegetation recovery rate and annual accumulation rate of community aboveground C, N, and P pools were used to evaluate the recovery rate of desert steppe ecosystems with different degradation levels after grazing exclusion, calculated using the following equations:

Annual vegetation recovery rate=$\frac{{N}_{Fi} - {N}_{Di}}{Grazing exclusion time }$

Annual accumulation rate of community aboveground nutrient pool = $\frac{{P}_{Fi} - {P}_{Di}}{Grazing exclusion time}$

where *N*_*Fi*_ indicates the community height, cover, and aboveground biomass after grazing exclusion; *N*_*Di*_ indicates these indexes for the degraded desert steppe before grazing exclusion; *P*_*Fi*_ indicates community C, N, and P pools after grazing exclusion and *P*_*Di*_ indicates these indexes for the degraded desert steppe before grazing exclusion. In this study, the grazing exclusion time was six years.

Data was tested for normality and homogeneity of variance. A pared-samples t-test was employed to compare the community’s quantitative characteristics and nutrient concentrations of dominant species and soil before and after grazing exclusion. A one-way analysis of variance test was used to test the community’s quantitative characteristics and nutrient concentrations of dominant species and soil among the three degraded steppes after grazing exclusion. The multiple comparison method selected was the least significant difference (LSD). Structural equation modeling (SEM) was used to reveal the critical ecological processes of vegetation restoration in the degraded desert steppe. An initial model was established based on the current knowledge of the factors impacting vegetation restoration in degraded grassland ecosystems. In this study, we hypothesized: (1) a grassland’s degradation level directly affects community height, cover, and aboveground biomass; (2) vegetation restoration accelerates the accumulation of a community’s aboveground nutrient pool in degraded grasslands; (3) vegetation restoration drives nutrient transfer from the soil to the plant community, which decreases soil nutrients. The maximum likelihood method was used to construct the SEM model and the quality of the final model was evaluated using chi-squared values, *p* values, AIC, RMSEA, and CFI. Data analysis was performed using SPSS 24.0 (SPSS Inc., Chicago, IL, USA) and Amos 24.0 (Amos Development Co., Greene, Maine, USA).

## Results

### Effects of grazing exclusion on plant nutrients

**Fig. 2:**
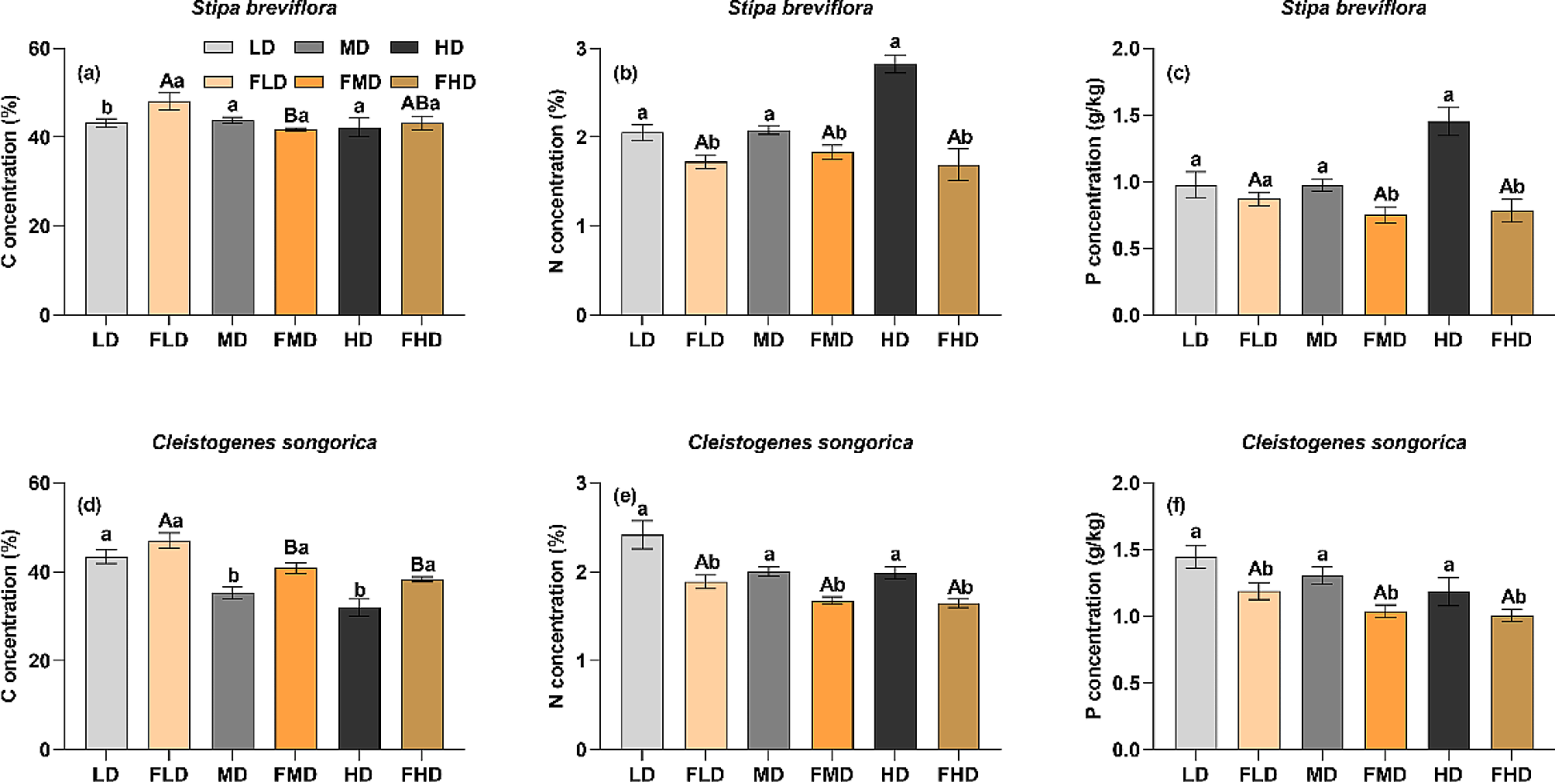
Nutrient concentrations of plant shoots for each degradation level after grazing exclusion. LD: light degradation; FLD: light degradation after fencing; MD: moderate degradation; FMD: moderate degradation after fencing; HD: heavy degradation; FHD: heavy degradation after fencing. The lowercase and uppercase letters indicate significant differences before and after grazing exclusion and among the three degradation levels after grazing exclusion at the 0.05 significance level, respectively

At the species level, the effects of grazing exclusion on plant nutrients differed depending on the level of degradation. Specifically, grazing exclusion significantly increased C concentration in plant shoots of *C. songorica* in the moderately and heavily degraded desert steppes (Fig. 2; *p* < 0.05), but had no significant effect on it in the lightly degraded desert steppe. Conversely, grazing exclusion remarkably increased C concentrations in *S. breviflora* in the lightly degraded desert steppe, but not in the moderately and heavily degraded desert steppes. Plant shoot N and P concentrations for both species were significantly reduced in the three degraded desert steppes after grazing exclusion (Fig. 2; *p* < 0.05). Interestingly, N and P concentrations in plant shoots of *S. breviflora* and *C. songorica* were not significantly different among the three degraded desert steppes after grazing exclusion (Fig. 2).

**Fig. 3:**
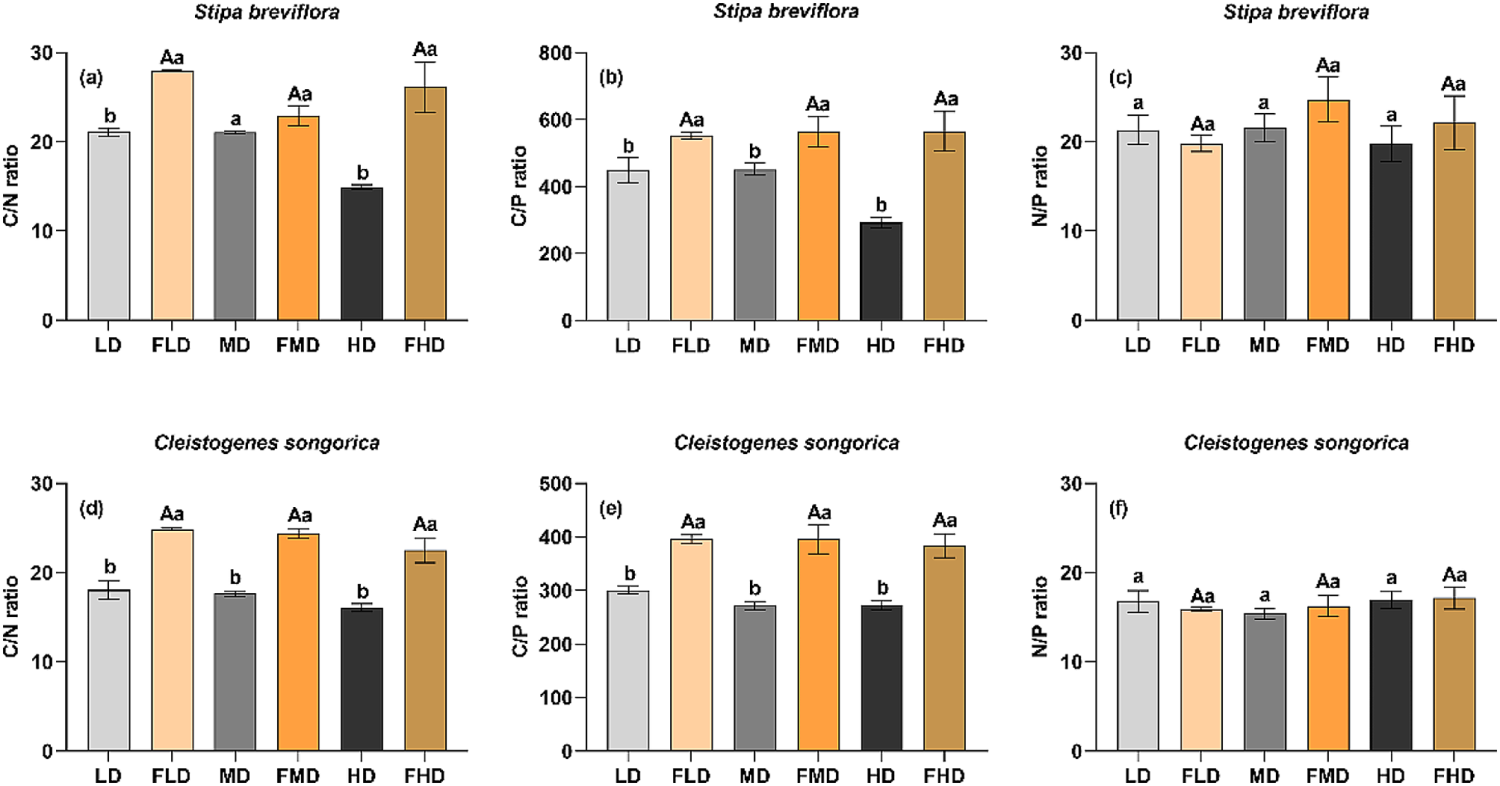
Plant shoot C, N, and P stoichiometry for each degradation level after grazing exclusion. LD: light degradation; FLD: light degradation after fencing; MD: moderate degradation; FMD: moderate degradation after fencing; HD: heavy degradation; FHD: heavy degradation after fencing. The different lowercase and uppercase letters indicate significant differences before and after grazing exclusion and among the three degradation levels after grazing exclusion at the 0.05 significance level, respectively

Plant shoot C, N, and P stoichiometry in the three levels of degraded desert steppes exhibited different responses to grazing exclusion. For the two main plant species, plant shoot C/N and C/P ratios significantly increased in all three degraded desert steppes after grazing exclusion (Fig. 3; *p* < 0.05), while the N/P ratio remained unchanged. Notably, *C. songorica* had a lower N/P ratio compared to *S. breviflora*. Importantly, plant shoot C/N, C/P, and N/P ratios of the two species did not differ among the three degraded desert steppes after grazing exclusion (Fig. 3).

**Fig. 4:**
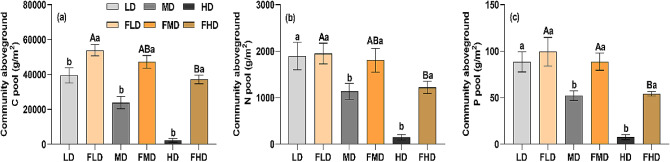
Community aboveground nutrient pools for each degradation level after grazing exclusion. LD: light degradation; FLD: light degradation after fencing; MD: moderate degradation; FMD: moderate degradation after fencing; HD: heavy degradation; FHD: heavy degradation after fencing. The different lowercase and uppercase letters indicate significant differences before and after grazing exclusion and among the three degradation levels after grazing exclusion grasslands at the 0.05 significance level, respectively

At the community level, grazing exclusion affected the community aboveground nutrient pools differently in the three levels of degraded desert steppes. Community aboveground C pool increased significantly in all three degraded steppes after grazing exclusion (Fig. 4; *p* < 0.05). Grazing exclusion had no significant effects on the community aboveground N and P pools in the lightly degraded desert steppe, but it remarkably increased these in the moderately and heavily degraded desert steppes (Fig. 4; *p* < 0.05). Moreover, the community aboveground nutrient pools in the heavily degraded desert steppe were significantly lower than those in the lightly and moderately degraded desert steppes (Fig. 4). Heavily degraded desert steppe had higher accumulation rate of community aboveground nutrient pools than the lightly and moderately degraded desert steppes (Table 2; *p* < 0.05). Compared with the N and P pools, the C pool in the heavily degraded steppe was more sensitive to grazing exclusion (Table 2).

**Table 2:** Annual accumulation dynamics of community aboveground nutrient pools for each degradation level after grazing exclusion

Experimental treatments	Accumulation rate of community aboveground C pool	Accumulation rate of community aboveground N pool	Accumulation rate of community aboveground P pool
	(g/m^2^ year)	(g/m^2^ year)	(g/m^2^ year)
FLD	3165.7 ± 41.6B	73.7 ± 15.8 C	0.7 ± 0.4B
FMD	3899.3 ± 298.8B	111.8 ± 14.8B	6.1 ± 1.8 A
FHD	6145.2 ± 310.0 A	178.7 ± 25.0 A	7.8 ± 0.8 A

*Note* FLD: light degradation after fencing; FMD: moderate degradation after fencing; FHD heavy degradation after fencing. The uppercase letters indicate significant differences among the three degradation levels after grazing exclusion at the 0.05 significance level, respectively

### Effects of grazing exclusion on plant community

Grazing exclusion had positive effects on vegetation restoration at both the species and community levels in each level of degraded desert steppe. At the species level, grazing exclusion significantly increased the aboveground biomass of *S. breviflora* and *C. songorica* in all three degraded desert steppes (Table 3; *p* < 0.05). However, after grazing exclusion, the aboveground biomass of *S. breviflora* in the heavily degraded desert steppe was remarkably lower than that in the lightly and moderately degraded desert steppes (Table 3; *p* < 0.05). In contrast, the aboveground biomass of *C. songorica* had did not differ significantly among the three levels of degradation after grazing exclusion (Table 3).

Similarly, grazing exclusion significantly increased the community height, cover, and aboveground biomass in all three degraded desert steppes, but these traits were the lowest in the heavily degraded desert steppe (Table 3; *p* < 0.05). The vegetation recovery rate after grazing exclusion differed depending on the level of degradation. The heavily degraded steppe had a higher restoration rate of community height, cover, and aboveground biomass than the moderately and lightly degraded desert steppes (Table 4).

**Table 3:** Quantitative characteristics of species and community for each degradation level after grazing exclusion

Experimental treatments	Aboveground biomass of *Stipa breviflora* (g/m^2^)	Aboveground biomass of *Cleistogenes songorica* (g/m^2^)	Aboveground biomass of other species (g/m^2^)	Community height (cm)	Community cover (%)	Community aboveground biomass (g/m^2^)
LD	91.9 ± 7.29b	7.01 ± 1.58a	4.26 ± 2.24b	9.9 ± 0.4b	30.8 ± 0.8b	103.16 ± 5.03b
FLD	112.88 ± 7.02aA	9.83 ± 2.80aA	19.91 ± 6.07aA	14.5 ± 0.7aA	46.1 ± 0.9aA	142.62 ± 12.02aA
MD	54.53 ± 4.50b	9.34 ± 2.03b	0.69 ± 0.23b	6.3 ± 0.3b	24.8 ± 0.6b	64.55 ± 3.45b
FMD	102.56 ± 10.53aA	17.74 ± 4.26aA	9.35 ± 3.00aB	13.4 ± 0.6aA	39.5 ± 1.4aB	129.65 ± 5.69aA
HD	5.44 ± 1.17b	5.63 ± 1.08b	0.65 ± 0.15b	1.7 ± 0.1b	13.2 ± 1.0b	11.72 ± 1.27b
FHD	85.96 ± 3.57aB	11.64 ± 2.47aA	1.20 ± 0.49aC	9.6 ± 0.5aB	33.9 ± 1.5aC	98.81 ± 4.69aB

*Note* LD: light degradation; FLD: light degradation after fencing; MD: moderate degradation; FMD: moderate degradation after fencing; HD: heavy degradation; FHD: heavy degradation after fencing. The lowercase and uppercase letters indicate significant differences before and after grazing exclusion and among the three degradation levels after grazing exclusion at the 0.05 significance level, respectively

**Table 4:** Annual vegetation restoration dynamics of the plant community for each degradation level after grazing exclusion

Experimental treatments	Increasing rate of aboveground biomass of *Stipa breviflora*	Increasing rate of aboveground biomass of *Cleistogenes songorica*	Increasing rate of community height	Increasing rate of community cover	Increasing rate of community aboveground biomass
	(g/m^2^ year)	(g/m^2^ year)	(cm/year)	(%/year)	(g/m^2^ year)
FLD	3.50 ± 2.84 C	1.01 ± 0.43B	0.8 ± 0.1B	2.6 ± 0.2B	6.58 ± 3.81 C
FMD	8.01 ± 2.53B	0.80 ± 0.10B	1.2 ± 0.1 A	2.5 ± 0.2B	10.85 ± 0.77B
FHD	13.42 ± 0.76 A	1.46 ± 0.27 A	1.3 ± 0.1 A	3.5 ± 0.2 A	14.52 ± 0.70 A

*Note* FLD: light degradation after fencing; FMD: moderate degradation after fencing; FHD: heavy degradation after fencing. The uppercase letters indicate significant differences among the three degradation levels after grazing exclusion at the 0.05 significance level, respectively

### Effects of grazing exclusion on soil nutrients

**Fig. 5:**
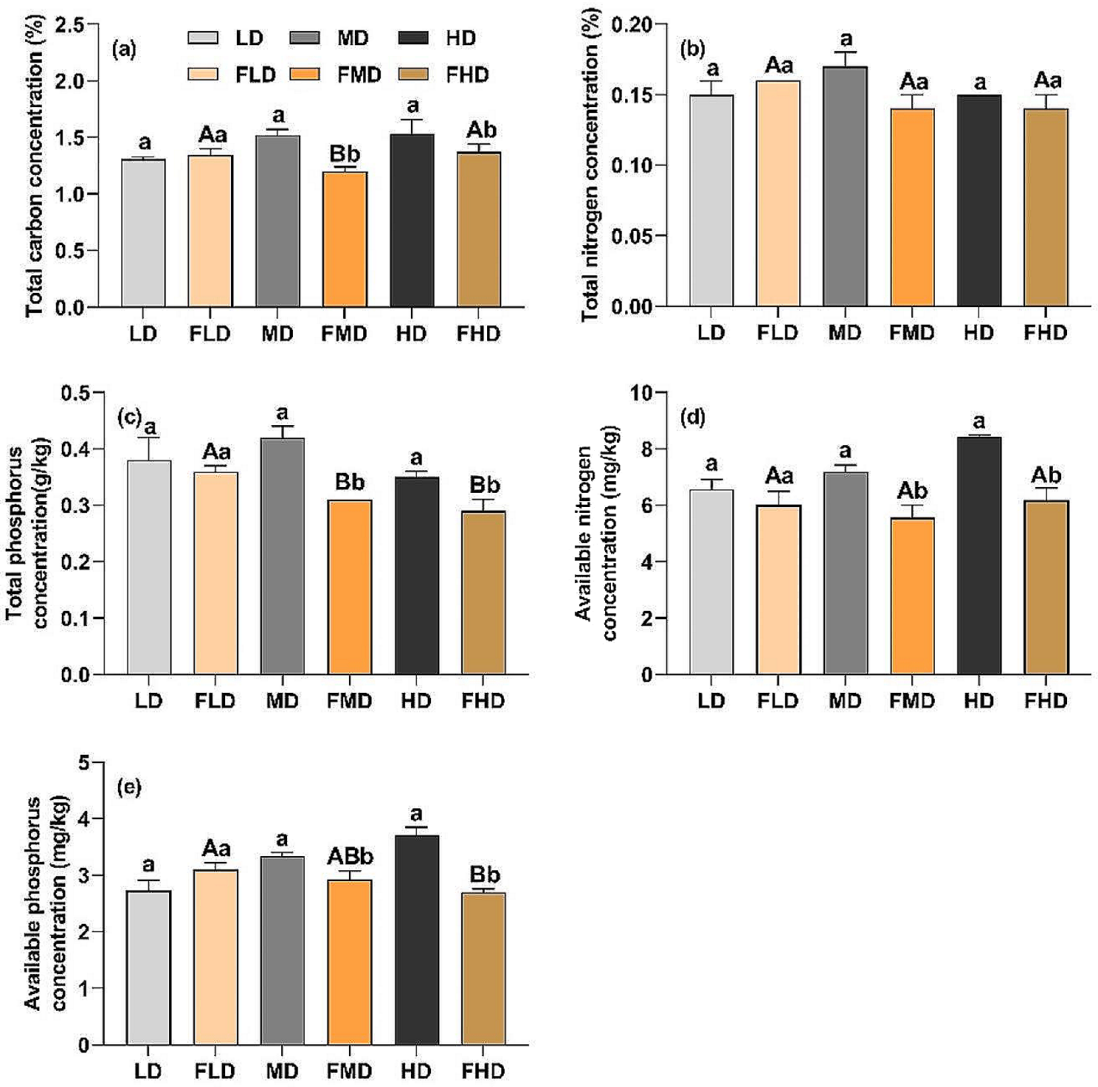
Soil nutrient concentrations for each degradation level after grazing exclusion. LD: light degradation; FLD: light degradation after fencing; MD: moderate degradation; FMD: moderate degradation after fencing; HD: heavy degradation; FHD: heavy degradation after fencing. The lowercase and uppercase letters indicate significant differences before and after grazing exclusion and among the three degradation levels after grazing exclusion at the 0.05 significance level, respectively

The response of soil nutrients to grazing exclusion varied depending on the level of degradation. In the lightly degraded desert steppe, grazing exclusion had no significant effects on soil total C, total N, total P, available N, or available P concentrations (Fig. 5). However, in the moderately and heavily degraded desert steppes, all soil nutrient concentrations, except for soil total N, significantly decreased after grazing exclusion (Fig. 5; *p* < 0.05). Moreover, compared to the soil total nutrients, the soil available nutrients exhibited more sensitive responses to grazing exclusion in each level of degraded steppe (Fig. 5).

### Relationship between vegetation restoration and nutrient transfer

**Fig. 6:**
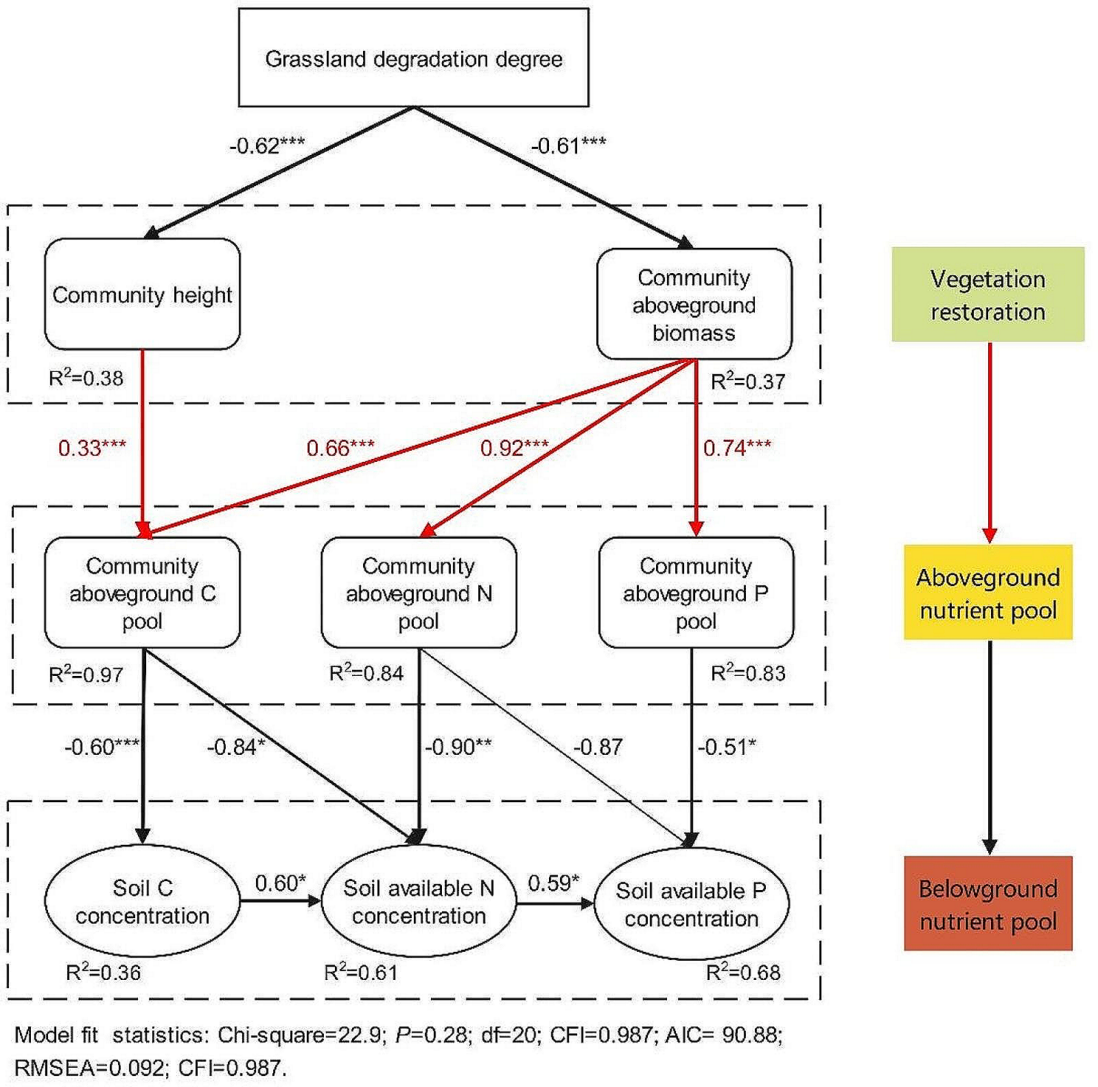
Relationship between nutrient transfer and vegetation restoration in the three levels of degraded desert steppe after grazing exclusion. The arrow directions connecting the boxes indicate the direction of causation. The width of the arrows indicates the strength of the path coefficients. Red and black arrows represent positive and negative correlations between the two measured variables, respectively. R^2^ indicates the proportion of variance explained. Values associated with solid arrows represent standardized path coefficients. *p < 0.05, **p < 0.01, and ***p < 0.001 indicate significance levels between two variables. Non-significant relationships are not shown

SEM analysis resulted in a well-fitting model that showed the relationships among relevant variables (*p* = 0.28; RMSEA = 0.092; Fig. 6). The grassland degradation level directly determined vegetation restoration in the degraded desert steppe. Community height and aboveground biomass explain 97%, 84%, and 83% of the total variance in community aboveground C, N, and P pools, respectively (Fig. 6). Community aboveground biomass was an important factor controlling the accumulation of community aboveground nutrient pools. Changes in community aboveground nutrient pool indirectly altered soil nutrient concentrations and these soil nutrients have coupling effects (Fig. 6). Moreover, grassland degradation levels indirectly altered community aboveground and belowground nutrient pools, accelerating nutrient transfer from the soil to the plant community.

## Discussion

### Effects of grazing exclusion on plant community and soil nutrients

The plant community and soil are crucial components of grassland ecosystems and exhibit unique responses to grazing exclusion, which can comprehensively alter these ecosystem’s function and process. We found that grazing exclusion had a positive impact on vegetation restoration in desert steppes with varying degradation levels, consistent with previous studies [21, 24, 30]. Grazing exclusion put an end to foraging and trampling by livestock, which resulted in an increase in height, cover, and aboveground biomass at both the species and community levels. Notably, among the desert steppes with varying degradation levels, it was the most heavily degraded steppe that had the highest vegetation restoration rate, which was inconsistent with previous studies [3]. Liu et al. found that fencing promoted vegetation restoration more in lightly degraded alpine meadows in the Tibetan Plateau, compared to heavily degraded ones, and they emphasized the importance of higher precipitation (about 700 mm) [3]. Precipitation played an important role in the vegetation restoration of fenced grasslands, which were more effective in semi-humid regions than in arid and semi-arid areas [31]. However, the precipitation in this study region is about 220 mm, and the soil available nutrients are poor (Fig. 5). The species composition included some cold-tolerant and drought-tolerant grasses, which were always facing drought stress [32]. Therefore, the plants in the heavily degraded desert steppe were more adaptable to disturbance, which may have contributed to their stronger compensatory growth [33], thereby decreasing the negative effects of heavy disturbance on plants at multiple levels. Thus, the heavily degraded desert steppe had the highest vegetation restoration rate in our study. However, despite this, the community height, cover, and aboveground biomass were significantly lower than those in the lightly or moderately degraded desert steppes, in line with previous studies [34]. This result indicates that vegetation restoration in response to grazing exclusion is dependent on the previous level of degradation. The importance of degradation level to vegetation restoration in grassland ecosystems may even be higher than that of grassland type, fencing year, and the grazing livestock. Therefore, fencing policies should be implemented scientifically in a way dependent on the grassland degradation level, and management strategies of fencing large areas should be avoided [24, 35].

Grazing exclusion also has direct or indirect effects on soil nutrient dynamics [13, 36]. In this study, soil nutrient concentrations in the moderately and heavily degraded desert steppes decreased remarkably after grazing exclusion, which was inconsistent with previous studies in alpine meadows, meadow steppes, and typical steppes [37]. It is believed that this is primarily due to differences in accumulation and decomposition of plant litter. The extremely low aboveground productivity resulted in the lowest litter accumulation in the desert steppe after grazing exclusion among different grasslands [10, 38]. The windy and less rainy climate also inhibited the activity of soil microorganisms [39], which reduced the litter decomposition rate. Furthermore, once grazing exclusion was implemented, the cessation of livestock feces and urine reduced the input of soil nutrients. More importantly, the rapid vegetation restoration after grazing exclusion caused the transfer of nutrients from the soil to the plant community, especially in heavily degraded desert steppe. Interestingly, some studies in alpine desert steppes and deserts have found similar results, that is, that fencing significantly decreased soil nutrient concentrations [16, 40]. Similarly, these alpine desert steppes and deserts had lower community aboveground productivity, so after grazing exclusion, there was little accumulation of plant litter. However, soil nutrient concentrations in the lightly degraded desert steppe did not change significantly after grazing exclusion, indicating that grazing exclusion affected the grassland’s soil nutrients differently depending on the level of degradation. These divergent results highlight the complex effects of grazing exclusion on soil nutrient dynamics. Thus, of the reason for mixed findings about how soil nutrients are affected by grazing exclusion could be driven by varying levels of grassland degradation in previous studies [3, 34]. The asymmetric responses of soil nutrients and the plant community to grazing exclusion in the different levels of degraded desert steppes had impacts on soil-plant nutrient cycling, which might drive changes in ecosystem structure and functions.

### Effects of grazing exclusion on plant nutrients

The sensitive and rapid response of plant nutrients to changes in grassland management is important for revealing soil-plant nutrient cycling. We found that grazing exclusion significantly reduced N and P concentrations but increased C/N and C/P of plant shoots in the grasslands at all degradation levels, but especially in heavily degraded ones, indicating that the growth rate of plants was slowed down after grazing exclusion. This result was consistent with some previous studies [14, 25]. The cessation of foraging by livestock slowed the growth rate of plants after grazing exclusion, which in turn reduced the demand for protein and nucleic acid [33]. Additionally, the decrease in soil nutrients also contributed to this phenomenon [41]. The changes in plant nutrients in after grazing exclusion in degraded grasslands also has profound effects on the community nutrient pool. This study found that the community aboveground nutrient pools in the degraded grasslands increased significantly after grazing exclusion, which was consistent with previous studies [42]. This is mainly related to the rapid vegetation restoration in the degraded desert steppes [18]. Furthermore, although the community aboveground nutrient pools in the heavily degraded grassland accumulated more quickly after grazing exclusion, its community nutrient pools were the lowest among the three levels of degraded grasslands, emphasizing that the vegetation status of degraded grasslands playes a critical role in controlling nutrient transfer. These results also suggested that plants might adopt different nutrient strategies at species and community levels in response to grazing exclusion.

Unlike for C, there were no significant differences in N and P concentrations, C/P, and C/N of plant shoots among the three levels of degraded grasslands after grazing exclusion, indicating that plants might use a similar nutrient strategy to adapt to grazing exclusion across varying levels of degradation. During restoration succession, (1) different plants might use the same nutrient strategy to adapt to grazing exclusion, resulting in the gradual recovery of the vegetation. This ecological process may not be related to the grassland degradation level. (2) Previous studies have shown that there was an “inflection point” in the restoration of degraded grasslands [3]. This might allow plants of different degraded grasslands to adapt to different nutrient strategies at the beginning of the grazing exclusion, and then later to adopt convergent nutrient adaptation strategies. As the vegetation recovered, the nutrient strategies of these plants gradually converged. In this study, the sample collection was conducted grasslands that had been fenced for six years, which may have exceeded the “inflection point” of nutrient strategy regulation among plants in these various degraded desert grasslands. Future studies should attempt to test these two hypotheses.

### Divergent effects of grazing exclusion on nutrient transfer

Vegetation restoration after grazing exclusion is closely interrelated with plant and soil nutrients, altering the nutrient transfer of grassland ecosystems. The community characteristics and soil and plant nutrient states responded differently to grazing exclusion across the three levels of degradation, indicating that grassland degradation level has a critical impact on the restoration of degraded grassland ecosystems. The SEM model indicated that grassland degradation level directly altered the community characteristics and nutrient accumulation of the community aboveground pool, which indirectly changed the nutrient transfer of grassland ecosystems. It also demonstrated that community aboveground biomass was a critical factor in regulating nutrient transfer from belowground to aboveground. The recovery rate of the grassland ecosystem (i.e., aboveground biomass and nutrient pool) in the heavily degraded desert steppe was the highest among the three steppes, indicating that the highest amount of nutrients were transferred from the soil to the plant community. This may be due to the severe destruction of vegetation in the heavily degraded desert steppe, resulting in an imbalance of nutrients between belowground and aboveground [43]. After grazing exclusion, the ecosystem regulates the nutrient transfer through its self-healing capacities and restores the nutrient balance between belowground and aboveground [43, 44]. This may be an important nutrient regulation mechanism for restoring degraded desert grassland ecosystems. This also proves that fencing-driven recovery of degraded grassland vegetation causes a transfer of ecosystem nutrients (i.e., a transfer of nutrients from soil to above-ground vegetation), supporting previous results from grassland management [43].

## Conclusions

Grazing exclusion is an effective technique for restoring degraded grassland ecosystems, as it drives vegetation restoration and changes the nutrient cycling of soil-plant systems. Grassland degradation level is a critical factor modulating these ecological processes. At the species level, plants in desert steppe used the same nutrient strategy across degradation levels by reducing nutrient concentrations to adapt to grazing exclusion. However, at the community level, grazing exclusion accelerated vegetation restoration of desert steppe differently depending on the degradation level, especially affecting the community aboveground biomass. Vegetation restoration increased nutrient transfer from belowground to aboveground, resulting in a decrease in soil nutrient concentrations. Community aboveground biomass was a critical factor regulating this nutrient transfer in the degraded desert steppe. These ecological processes of plants and soil were more efficient in the heavily degraded desert steppe compared to less degraded ones.

## Data Availability

The data are available from the corresponding author.
